# Curvature recognition and force generation in phagocytosis

**DOI:** 10.1186/1741-7007-8-154

**Published:** 2010-12-29

**Authors:** Margaret Clarke, Ulrike Engel, Jennifer Giorgione, Annette Müller-Taubenberger, Jana Prassler, Douwe Veltman, Günther Gerisch

**Affiliations:** 1 Program in Genetic Models of Disease, Oklahoma Medical Research Foundation, Oklahoma City, OK 73121, USA; 2 Nikon Imaging Center at the University of Heidelberg, Bioquant, 69120 Heidelberg, Germany; 3 Institut für Zellbiologie, Ludwig-Maximilians-Universität München, 80336 München, Germany; 4 Max-Planck-Institut für Biochemie, 82152 Martinsried, Germany; 5 Beatson Institute for Cancer Research, Glasgow G61 1BD, UK

## Abstract

**Background:**

The uptake of particles by actin-powered invagination of the plasma membrane is common to protozoa and to phagocytes involved in the immune response of higher organisms. The question addressed here is how a phagocyte may use geometric cues to optimize force generation for the uptake of a particle. We survey mechanisms that enable a phagocyte to remodel actin organization in response to particles of complex shape.

**Results:**

Using particles that consist of two lobes separated by a neck, we found that *Dictyostelium *cells transmit signals concerning the curvature of a surface to the actin system underlying the plasma membrane. Force applied to a concave region can divide a particle in two, allowing engulfment of the portion first encountered. The phagosome membrane that is bent around the concave region is marked by a protein containing an inverse Bin-Amphiphysin-Rvs (I-BAR) domain in combination with an Src homology (SH3) domain, similar to mammalian insulin receptor tyrosine kinase substrate p53. Regulatory proteins enable the phagocyte to switch activities within seconds in response to particle shape. Ras, an inducer of actin polymerization, is activated along the cup surface. Coronin, which limits the lifetime of actin structures, is reversibly recruited to the cup, reflecting a program of actin depolymerization. The various forms of myosin-I are candidate motor proteins for force generation in particle uptake, whereas myosin-II is engaged only in retracting a phagocytic cup after a switch to particle release. Thus, the constriction of a phagocytic cup differs from the contraction of a cleavage furrow in mitosis.

**Conclusions:**

Phagocytes scan a particle surface for convex and concave regions. By modulating the spatiotemporal pattern of actin organization, they are capable of switching between different modes of interaction with a particle, either arresting at a concave region and applying force in an attempt to sever the particle there, or extending the cup along the particle surface to identify the very end of the object to be ingested. Our data illustrate the flexibility of regulatory mechanisms that are at the phagocyte's disposal in exploring an environment of irregular geometry.

## Introduction

Phagocytes, as macrophages, neutrophils or *Dictyostelium *cells, respond to the shape of surfaces they encounter. These cells are capable of moving on flat surfaces to which they adhere. However, when exposed to a three-dimensional particle such as a bacterium or yeast, a phagocyte forms a circular extension, the phagocytic cup, which progressively encloses the particle. At the end of uptake, the cup closes on top of the particle by membrane separation and fusion. In this way, the inner surface of the cup becomes the phagosome membrane encaging the particle, and the outer surface remains an integral part of the plasma membrane surrounding the entire cell.

Phagocytosis requires forces that act against cortical tension, which increases with expansion of the cell-surface area [1]. Actin polymerization at the edge of the phagocytic cup drives protrusion and mediates the contractile activity that is responsible for closing the cup on top of the particle. This contractile activity has been illustrated by pairs of macrophages attempting to engulf a single erythrocyte [2]. The phagocytes squeezed the erythrocyte, pulling it into a string surrounded by protrusions from the two cells. Myosin-IC was the only myosin detected in the protrusions that surrounded the connecting string.

Phagocytes accommodate themselves not only to the size but also to the shape of a particle. This was demonstrated by Champion and Mitragotri [3], who exposed macrophages to non-spherical polystyrene particles of controlled shape. Depending on the local angle at the point of attachment, the phagocytes either engulfed an elliptical disc or spread along its flat surface. A 'UFO'-shaped particle was internalized when the phagocytes attached to the convex dome or ring region, but not when they attached to the concave region between these. Another property to which phagocytes can respond is the rigidity of the prey. This response involves mechanosensing, which in macrophages depends on Rac1-mediated signal transduction [4].

In this study, we used living budding yeast as rigid particles, analogous to those to which *Dictyostelium *cells are exposed in their natural habitat. *Dictyostelium *cells rely primarily on the physical properties of hydrophobic or slightly hydrophilic surfaces for the uptake of a particle. To such surfaces, the cells attach via a variety of plasma membrane proteins [5,6]. Although no specific receptor-ligand interaction is required for *Dictyostelium *cells to engulf a particle such as a latex bead, these cells do respond to certain surface-bound carbohydrates [7-9]. The molecular machinery for transmembrane signaling to the actin cytoskeleton is advanced in *Dictyostelium *cells and comparable with that established in mammalian phagocytes. Heterotrimeric G proteins are essential for local activation of the actin system beneath an attached particle [10] and conserved actin-binding proteins, such as talin, are involved in cell-particle adhesion [11].

Proteins that associate with actin in phagocytic cups include myosin-IB (MyoB) [12], myosin-IK [13,14], myosin-VII [15], the Arp2/3 complex [16] and coronin [17-20]. The three myosins harbor lipid-binding sites or farnesyl residues for anchorage to the membrane of the incipient phagosome. The Arp2/3 complex colocalizes with filamentous actin consistent with its role in nucleating branched actin assemblies. The *Dictyostelium *coronin CorA is involved in destabilizing actin structures [21,22]. It is missing at the very rim of the extending cup and is otherwise recruited remote from the phagosome membrane to a layer at the cytoplasmic face of the network of actin filaments [20].

As long as a phagocyte takes up small free-floating particles, it can easily recognize the site at which the cup should close behind the particle. However, if particles are of irregular shape or are attached to another surface, the phagocyte must identify the end of the entity that it is seeking to ingest. A specific receptor-ligand recognition mechanism such as that provided by the antibody-mediated F_c_-receptor system of macrophages is not suitable for free-living phagocytes, which use microbes with varying surface properties as nutrients. Curvature sensing provides these phagocytes with a more versatile mechanism to define the boundary of a particle.

To investigate the role of curvature sensing in phagocytosis, we used particles with a constriction separating two convex portions of their surface. Specifically, we employed a mutant of *Saccharomyces cerevisiae *arrested at intermediate stages of bud formation, which has proved to be a useful tool for the study of endocytic trafficking [23,14]. When a phagocyte takes up one of these particles, it faces a conflict. It must decide whether to treat the concave neck between the mother and bud as the end of the particle or to continue to search along the surface of the particle for the actual end. Thus, the phagocytic cup may stop at the negative curvature of the neck or it may extend beyond the neck until the entire particle has been engulfed.

Outward-curving membranes, such as those surrounding the concave neck of a particle, can be recognized and stabilized by I-BAR domains [24,25]. The *D. discoideum *genome contains a single gene (DDB_G0274805) encoding a protein that contains an I-BAR domain [26]. This protein, called IBARa (IbrA), also contains an SH3 domain, identifying it as a member of the insulin receptor tyrosine kinase substrate p53 (IRSp53) subfamily. SH3 domains and other protein-protein interaction surfaces allow IRSp53-like proteins to recruit proteins that modulate actin dynamics such as Rac, neuronal Wiskott-Aldrich syndrome protein (N-WASP) and the WASP family verprolin homologous protein (WAVE) [26,27]. We investigated a green- fluorescent protein (GFP) fusion to IBARa as a potential curvature sensor and signal transducer to the actin system.

In this report we show that phagocytes not only sense the negative curvature of the neck as the putative end of the particle, but also apply force at the neck in an attempt to sever this linkage and close the cup. 'Biting' of entire cells into pieces is an action crucial to the pathogenicity of *Entamoeba histolytica*, which cuts off and ingests pieces of intestinal epithelial or liver parenchymal cells [28]. Our data indicate that cutting of semisolid structures into pieces is a general capacity of phagocytes. In this paper we focus attention on the dynamics of protein recruitment, which underlies the scanning of particle surfaces and allows the generation of force for phagosome closure.

## Results

### Actin accumulation and force generation at concave particle surfaces

**Figure 1:**
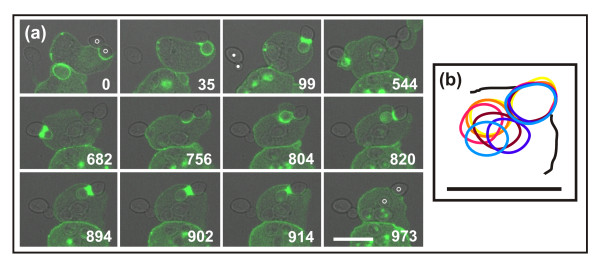
**Actin dynamics and force generation against a budded yeast particle**. The cell expresses LimEΔ- GFP to label filamentous actin. **(a) **Time series showing the actin label in green superimposed on the greyscale brightfield image. The cell interacts first with the budded yeast on the right (circles indicating mother cell and bud), strongly accumulating actin around its neck. While this particle is released, the particle on the left (dots) is attacked in the same manner, again unsuccessfully. A second attempt on the first particle leads to severing of the linkage between mother and bud. Time is indicated in seconds. The complete series is shown in Additional file 1. **(b) **Particle movement before and after the severing event. Positions of the two halves of the particle are indicated at 4 second intervals from yellow through red to blue starting at 883 s of the sequence in (a). The cell border is indicated as a black contour. Whereas the outer part of the particle stays in place, the internalized one is moved about within the cell after severing of the linkage. Bars = 10 μm.

To monitor the dynamics of actin accumulation around a particle consisting of convex and concave surfaces, we used *Dictyostelium *phagocytes expressing a label for filamentous actin, LimEΔ-GFP [29], and exposed the cells to particles of a yeast mutant arrested in budding, which causes the yeast to assume an hourglass shape. Figure 1 illustrates the engagement of a cell with two such particles. A prominent response is the strong accumulation of actin filaments around the neck between mother cell and bud. In the first two encounters, the uptake attempt stalls at the neck and the particle is eventually released. On the third attempt, the cell succeeds in severing one of the budded yeast and ingesting one of its portions (Additional file 1).

### Switching between two modes of interaction with a particle of complex shape

**Figure 2:**
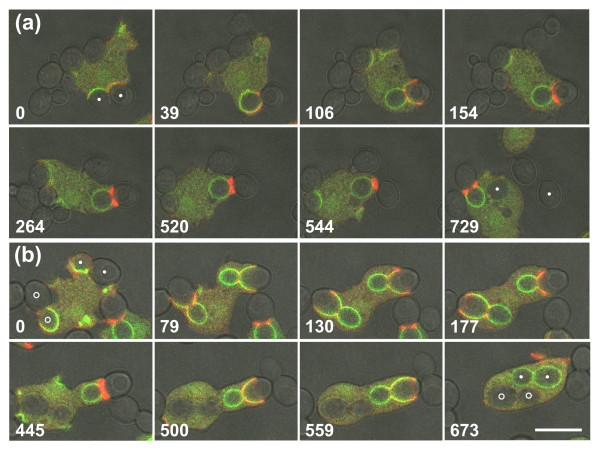
**Decision making in the phagocytic response to positive and negative curvature**. Fluorescence images of PHcrac-GFP (green) and mRFP-LimEΔ (red) are superimposed on greyscale brightfield images that show the shape of budded yeast particles. The two lobes of each particle are marked by dots or circles in the first and last frames of each series. Time is indicated in seconds. **(a) **Interplay of a *Dictyostelium *cell with a budded yeast, ending in severing of the particle. The cup extends halfway around the distal lobe of the particle (frame at 106 seconds) before retreating to the neck, where actin strongly accumulates (154 to 520 seconds). Finally, the particle is severed (544 to 729 seconds) and one lobe is internalized. **(b) **A similar sequence of events, but ending with complete uptake of the budded yeast labeled with dots. First, the cup extends up to part of the distal lobe (79 seconds). Subsequently the cup draws back to the neck of the particle (130 and 177 seconds) where actin strongly accumulates (445 seconds). Eventually the actin disassembles and the cup extends again (500 and 559 seconds), until the particle is completely engulfed (673 seconds). A third variant of interaction with a particle is exemplified by the budded yeast marked with circles at the left of the cell. Here the cup progresses continuously around the entire particle without a strong accumulation of actin at the neck. The complete series are shown in Additional files 2 and 3. Bar = 10 μm.

The membrane of a phagocytic cup is distinguished from the surrounding plasma membrane by the enrichment of PI(3,4,5)P3 (PIP3) [30,31]. To investigate the localized accumulation of actin in concert with the expansion and retraction of the entire cup, we double-labeled cells with monomeric red fluorescent protein (mRFP)-LimEΔ for filamentous actin and with PHcrac-GFP, which recognizes PIP3 together with its degradation product PI(3,4)P2. Figure 2 illustrates the distribution of the PHcrac label during the uptake of budded yeast particles. Figure 2a shows an uptake attempt that ends with separation of the two lobes of the particle, and Figure 2b a cell completing the uptake of two budded yeast. These outcomes illustrate the two options a phagocyte has: to proceed with extension of the phagocytic cup along the convex surface of the particle in search of the particle's end, or to concentrate actin around a concave zone of the particle in an attempt to separate it into pieces and engulf the portion already internalized (Additional files 2 and 3). The treatment of the upper yeast in Figure 2b reveals that a cell can switch from one strategy to the other. The cell initially extends the cup beyond the neck of the particle (frames 79 and 130), then retracts the cup back to the neck (frames 177 and 445), and finally extends the cup again to complete uptake (frames 500, 559 and 673). Even after 4 minutes of continuous arrest at the neck, the actin suddenly disassembles there and the rim of the cup resumes its progress. This behavior indicates that attempts to ingest a particle of complex shape may lead to fluctuations of the phagocytic cup between two unstable states, either arresting at concave regions of the particle or progressing along its convex distal portion. If neither of the strategies leads to closing of the cup, the cell employs a third option: it disassembles actin and releases the particle (Figure 1).

### Decision-making in phagocytosis is based on a delicate balance of actin polymerization and depolymerization

The reversibility of actin polymerization around the particles (Figure 2) suggests that phagocytosis is under continuous positive and negative control up to the closure of the cup, and a slight shift from net polymerization of actin to net depolymerization results in the sudden release of a particle. To monitor the spatiotemporal pattern of actin polymerization dynamics in response to budded yeast, we combined an mRFP-LimEΔ label for filamentous actin with a GFP-coronin label. Because coronin (CorA), in concert with actin interacting protein 1 (Aip1), promotes the depolymerization of actin structures in *Dictyostelium *[22], it can be used as a marker for sites of actin disassembly, as described previously [32,19,33].

**Figure 3:**
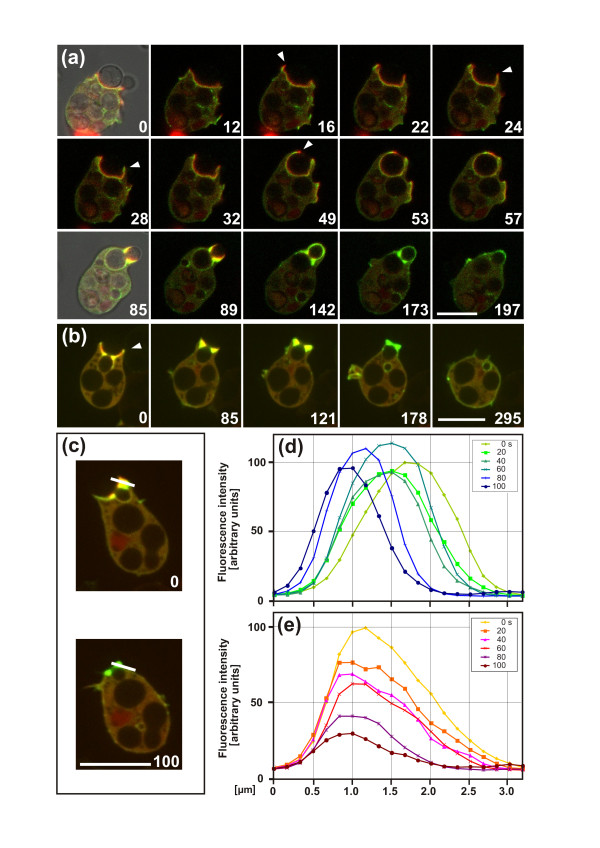
**Coronin dynamics in particle uptake and release**. Cells expressing GFP-coronin (green) and mRFP-LimEΔ (red) are exposed to budded yeast. **(a) **Complete uptake of budded yeast. In the frames at 0 and 85 seconds, brightfield images are superimposed on the fluorescence images to show particle shape. At 0 to 53 seconds, the extension of a cup occurs around the yeast mother cell, with a clear layering of coronin at the cytoplasmic face of the actin layer. During cup protrusion, actin precedes coronin at the edges of the cup (16 and 49 seconds, arrowheads). During expansion of the cup over the bud of the particle, the coronin label is again layered on the cytoplasmic face of the actin label (85 and 89 seconds). Subsequently, actin is disassembled (142 and 173 seconds), and finally coronin is dispersed upon closure of the cup (197 seconds). At stages of local retraction, coronin transiently replaces the actin at the rim (24 and 28 seconds, arrowheads). The complete series is shown in Additional file 4. **(b) **Partial uptake followed by release of budded yeast. The release starts with the green coronin label becoming prominent at the edge of the cup (0 seconds, arrowhead), followed by stratified accumulation of actin and coronin at the neck (85 and 121 seconds). Actin disappears gradually before coronin (178 seconds) before retraction of the cup is finished and the particle released (295 seconds). **(c) **Positions of the line scan displayed in (d) and (e) that measured the dynamics of coronin relative to actin disassembly. The scan has a width of 3 pixels. **(d, e) **Fluorescence intensities of (D) GFP-coronin and (E) mRFP-LimEΔ at the neck of a budded particle before its release, measured at intervals of 20 seconds. The fluorescence intensities are normalized to the highest value in the first frame of each of the two channels, which is set to 100. The scan direction from the phagosome to the cytoplasm is plotted from left to right. Because the phagosome was moving during the run, the scan positions had to be slightly readjusted to the actual border of the cup. Bars = 10 μm.

In the progressing cup, coronin decorates the boundary between the actin layer and the cytoplasm remote from the phagosome membrane (Figure 3a, 0 to 49 second frames). One feature revealed by this recording of uptake is of particular interest. As long as the cup progresses, the actin label leads at the rim of the cup, and coronin is enriched in a zone behind (16 and 49 second frames). When the progression temporarily stops, the green coronin label becomes dominant at the very rim (28 second frame), to be replaced by the actin label as soon as the cup resumes extension (Additional file 4).

Because of the increased thickness of the actin layer in the neck region of the phagocytic cup, coronin dynamics during actin disassembly can be easily measured there. The scanning of fluorescence intensities shows that the coronin peak translocates across a distance of almost 2 μm toward the phagosome membrane while the actin is disassembled (Figure 3d, e).

### Turnover of filamentous actin structures determined by fluorescence recovery after photobleaching

The localization of coronin to the dense actin layer surrounding the neck of the particle suggests that this layer is not static but is subject to continuous turnover. Polymerization of actin along the cup surface is important for force generation, and depolymerization is essential for rapid switching between expansion and regression of the cup. To measure the turnover of actin filaments by fluorescence recovery after photobleaching (FRAP), we monitored the fluorescence of GFP-actin along transverse sections through the actin network that surrounds the concave region of a particle. The fluorescence recovery recorded reflects the incorporation of actin subunits into actin structures under the steric conditions of a phagocytic cup, which is a thin circular lamella connected at its base to the reservoir of diffusible actin subunits in the cell body.

**Figure 4:**
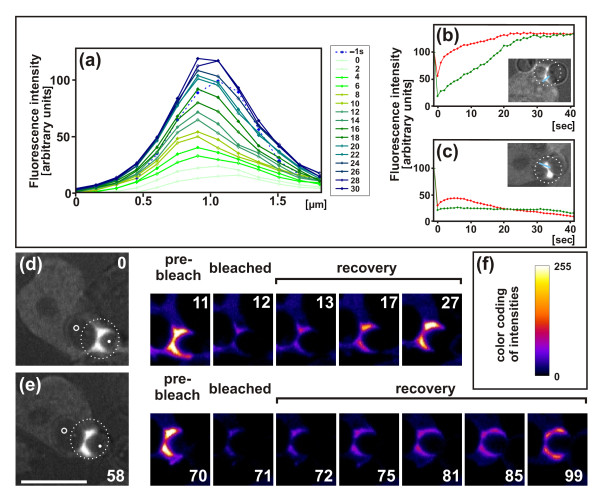
**Fluorescence recovery after photobleaching (FRAP)**. Cells expressing GFP-actin were fed with budded yeast. For the analysis shown in (b) and (c) the cells expressed also mRFP-LimEΔ. The bleached areas are circumscribed by circles. **(a) **Fluorescence intensities of GFP-actin at the neck of a particle from the phagosome membrane (left) to the cytoplasmic space (right). Fluorescence recovery after bleaching was plotted at 2 second intervals and its temporal succession indicated by color-coding the plots from light green to blue. The pre-bleach scan (-1 second) is demarcated by a dotted line. **(b) **Fluorescence intensities integrated over the areas shown in the plot of (a). In this case, recovery even exceeded the pre-bleach values. Time of the first image after a bleaching pulse was set to zero. **(c) **Plot of integrated fluorescence intensities similar to (b), but recorded at a stage of net disassembly of actin. After the bleaching pulse, the LimEΔ marker bound to the remaining actin structures although the incorporation of fluorescent actin subunits had ceased. For (a to c) fluorescence intensities were determined along slices = 3 pixels in width through the actin-rich zone that surrounds the concave neck of a particle. In (b, c) fluorescence intensities were normalized by setting the pre-bleach values to100, and scan positions are shown in blue within the insets. Green curves represent fluorescence intensities of GFP-actin, red curves of mRFP-LimEΔ. **(d, e) **A cell that switches from (d) arrest of the cup at the neck of the particle to (e) cup progression around the entire particle. Mother cell (circle) and bud (dot) are demarcated in the panels at 0 and 58 seconds showing brightfield illumination superimposed on the fluorescence images. The entire sequence is shown in Additional file 5. Time is indicated in seconds after the first frame of (d). The first bleaching pulse was set between the frames taken at 11 and 12 seconds while the cup was arrested, and the second pulse between 70 and 71 seconds after cup extension had resumed. Bar = 10 μm. **(f) **Look-up table showing color coding of the eight-bit intensity scale used in (d) and (e).

In Figure 4a, we show the evolution of fluorescence recovery along one of the optical slices. The fluorescence intensities integrated over a distance of 2 μm from the phagosome surface were plotted as a function of time (Figure 4b). The fluorescence recovery of GFP-actin was characterized by a nearly linear increase, indicating that the incorporation of new actin subunits proceeded with a constant velocity, until after about 25 seconds the bleached GFP-actin was completely replaced with newly incorporated fluorescent actin. This recovery kinetics differed from the fluorescence recovery of mRFP-LimEΔ, which displayed saturation kinetics and showed that diffusion into the bleached actin-rich area was sufficiently rapid for half-maximal binding within 3 seconds.

For comparison, recovery was also assayed before the release of a particle, in parallel with the coronin dynamics shown in Figure 3(d, e). No incorporation was detected in this context, indicating that actin polymerization was shut off while depolymerization ensued.

Figure 4(d, e) shows the fluorescence recoveries in a case similar to that in Figure 2b. First the cup was arrested for more than 1 minute at the neck region of the particle; subsequently the cup resumed extending until the particle was completely engulfed (Additional file 5). The first bleaching pulse was applied while the cup was arrested. Fluorescence recovery was already detectable within 1 second close to the neck of the particle, and within 15 seconds throughout the entire actin-rich area around the neck (Figure 4d). As this cup did not extend during the measurement, the fluorescence recovery reflects the treadmilling of actin under the steady-state condition of an arrested cup.

After the second bleaching pulse, the fluorescence recovered during a period of cup protrusion. Recovery was now detected along the extending cup (Figure 4e, frames at 71 to 99 seconds). At the neck region, the recovery was less intense than after the first bleaching event, consistent with the disassembly of actin at this region after a regulatory switch to cup protrusion (Figure 2b).

The FRAP data indicate that during progression of a phagocytic cup, actin is polymerized not only at the edge of the cup, but a region of intense actin polymerization is induced by the concave neck of a particle. Furthermore, actin is polymerized at sites distributed over convex regions of the phagosome, as revealed by the fluorescence recovery shown in Figure 4. These data prompted us to search for an activator of actin polymerization along the membrane of the incipient phagosome apart from its edge.

### Ras activity in phagocytosis

**Figure 5:**
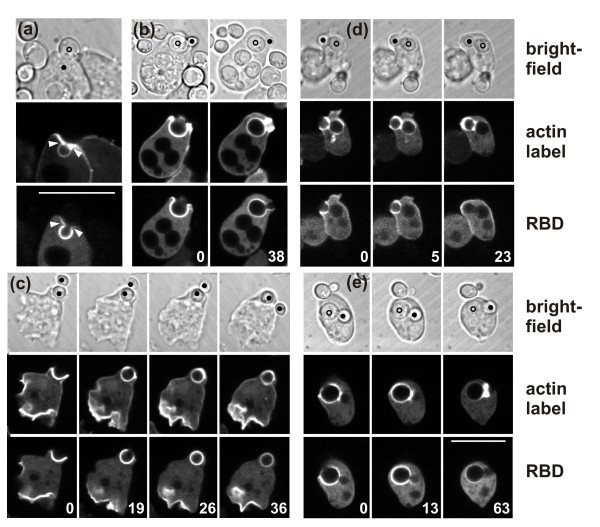
**Mapping of activated Ras relative to filamentous actin during the uptake of budded yeast**. Cells were double-labeled with GFP fusions of Ras-binding domains (RBDs) and mRFP-LimEΔ. The RBDs of (a-c) human Raf-1 and (d, e) *Dictyostelium *NdrC kinase were used. Top panels show brightfield images of phagocytes and yeast (marked by dots or circles), middle panels fluorescence images of filamentous actin labeled with mRFP-LimEΔ, and bottom panels the simultaneously recorded localization of RBDs. **(a) **A stage of partial uptake showing actin accumulation at the neck of the bud, which is not paralleled by an enrichment of activated Ras (arrowheads). **(b) **Two stages of complete uptake, showing strong increase in the actin label at the bud, whereas the RBD label at this site remains low. **(c) **Sequence showing severing of a particle. At the time the outer part of the particle is cleaved off (26 seconds), the site of separation is not discriminated by its Ras activity. **(d) **Three stages of a complete uptake. The actin is increasing around the neck of the particle when the RBD label is already fading out. **(e) **Uptake of a particle that is finally released. Within the sequence comprising 1 minute, actin strongly accumulates at the bud neck, whereas RBD shows strongest binding at 13 seconds around the large mother cell. Numbers in (b-e) indicate seconds after the first frame. Bars = 10 μm; the bar in (e) applies also to (b-d).

A candidate activator of actin polymerization in phagocytosis is Ras, which regulates actin assembly in motile *Dictyostelium *cells [34,35]. To probe for sites of Ras activation during phagocytosis, GFP-tagged domains that bind to activated Ras (RBDs) were co-expressed with mRFP-LimEΔ. Activated Ras was not specifically enriched at the edge of phagocytic cups (Figure 5). Instead, it was localized to the entire cup membrane including the cup's base. This pattern of Ras activation is consistent with actin polymerization at sites other than the rim of the cup. However, Ras was not more strongly activated in the zone of increased actin polymerization around the neck of a particle. Indeed, activated Ras appeared to be reduced at the strongly bent membrane. We therefore explored other factors that might produce a curvature-dependent signal for actin polymerization. These possibilities were 1) non-uniform distribution of specific phosphoinositides in the membrane of the phagosome, 2) cortical tension caused by local bending of the membrane, and 3) geometrical sensing of negative curvature by a protein harboring an I-BAR domain.

### Patterns of phosphoinositide recruitment in relation to actin accumulation

**Figure 6:**
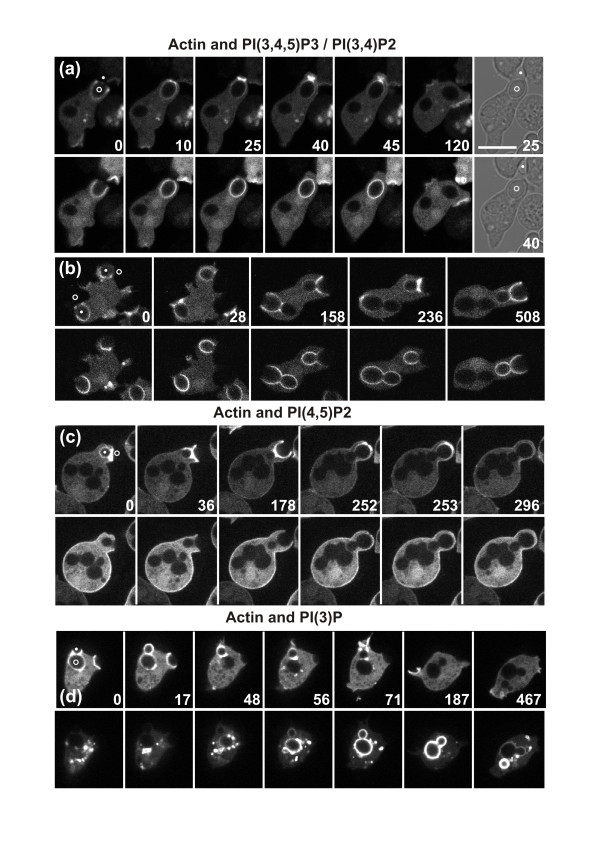
**Actin polymerization around budded yeast in relation to phosphoinositide localization**. The paired images show the mRFP-LimEΔ label for filamentous actin (upper row in each time series) and the GFP-tagged biosensors for specific phosphoinositides (bottom rows). **(a, b) **The PH domain of CRAC, which binds to phosphoinositides PIP3 and PI(3,4)P2; **(c) **the PH domain of PLCδ, which binds to PI(4,5)P2. **(d) **The 2FYVE domain, which binds to PI(3)P. Time is indicated in seconds. In the first frames, the mother cell of the yeast is indicated by a circle, the bud by a dot. In (a) the particle is severed; the 25 and 40 seconds frames are brightfield images showing separation of the mother cell and bud. In (b-d) the particles are completely taken up. Bar = 10 μm.

In the merged images of Figure 2 showing the actin label (red) overlaid on the PIP3 probe (green), the PIP3 signal does not appear brighter at the neck of the particle where actin is going to accumulate. This implies that the strong accumulation of actin at sites of negative curvature is not primed by an elevation in PIP3. To illustrate this, we show in Figure 6(a, b) the GFP and mRFP channels separately in two time series preceding the formation of an actin ring. These data demonstrate that PIP3 was not enriched in the region of high actin accumulation. As was true for activated Ras, PIP3 appeared to be depleted as actin began to accumulate at the neck of a particle (Figure 6a, 25 second frame; Figure 6b, 236 second frame).

In addition to PIP3, we examined two other phosphoinositides, PI(4,5)P2 (PIP2) and PI(3)P, which have distinct temporal patterns of localization and are known to act as signal transducers during the early stages of endocytosis [36]. In macrophages, PIP2 has been suggested to induce the recruitment of actin to the phagocytic cup [37]. Therefore, we monitored this phosphoinositide in the neck region of a budded yeast where actin was strongly accumulating. In *Dictyostelium*, PIP2 declined along the membrane of the incipient phagosome during extension of the cup, and around the neck region there was no exception to this overall reduction (Figure 6c). In human neutrophils, PIP2 was also found to decline during phagocytic cup formation [38].

PI(3)P is recognized as an ubiquitous marker of the early endosome stage [39]. Similarly, in *Dictyostelium*, this phosphoinositide was distinguished from the two other phosphoinositides by its appearance at the phagosome membrane only after the cup had closed (Figure 6d). PI(3)P did not exhibit precocious recruitment to the neck region of a budded yeast, so it cannot be responsible for demarcating sites of negative curvature on the particle. Thus, none of the phosphoinositides we assayed became enriched at the neck where actin was induced to accumulate.

### A curvature sensor recruited to the neck region

**Figure 7:**
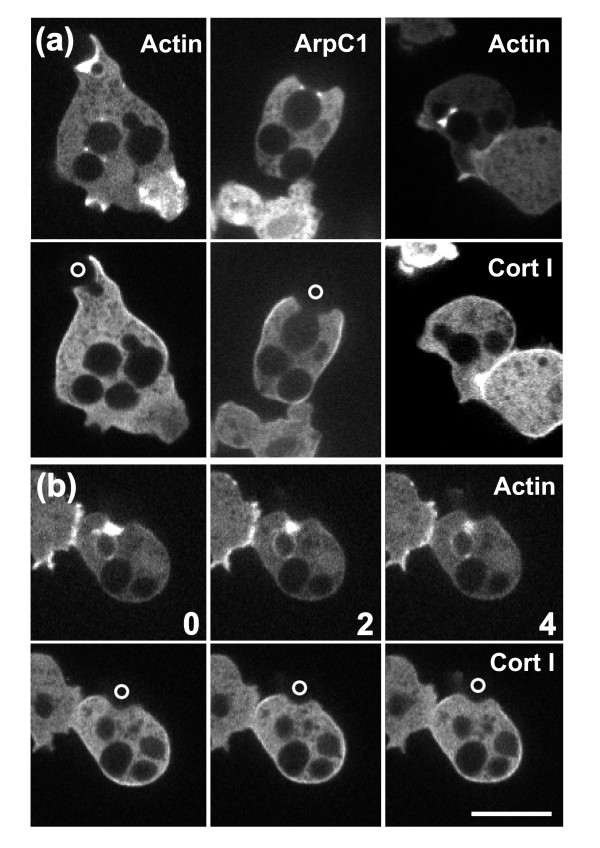
**Cortexillin depletion in phagocytic cups**. The external portions of the budded yeast, which are not visible in the fluorescence images, are denoted by circles. The cells are expressing mRFP-LimEΔ for actin or mRFP-ArpC1 for the Arp2/3 complex (upper panels) together with GFP-cortexillin I (lower panels). **(a) **Three examples of uptake of budded yeast: (left panel) an early stage of uptake, (middle) an intermediate stage, and (right) completed uptake. At all stages, cortexillin I is uniformly depleted from the phagosome membrane, whereas actin and the Arp2/3 complex are enriched around the neck of the particle. **(b) **Sequence showing severing of a budded yeast with strong accumulation of actin around the neck of the particle. Cortexillin I is depleted from the entire cup at all stages of severing. Numbers indicate time in seconds. Bar = 10 μm for all images.

We next explored whether the cells might be responding to alterations in cortical tension caused by acute bending of the membrane. If so, the bending stiffness of the cell cortex should be relevant for curvature recognition. To test this possibility, we examined the role of cortexillins I and II, a pair of actin bundling proteins that act as a heterodimer to modulate cortical stiffness [40,41]. In *Dictyostelium *mutants lacking both isoforms, the bending stiffness of the cell cortex is dramatically reduced, down to a value close to that of a free lipid bilayer [41]. Using GFP-tagged cortexillin I, we found that the association of this protein with the phagocytic cup was invariably reduced relative to the plasma membrane, not only in contact with the convex but also in contact with the concave regions of the particle (Figure 7). As cortexillin I harbors a PIP2-binding site [42], this finding is consistent with the reduced PIP2 content of the phagosome membrane seen in Figure 6c. The uniform reduction of cortexillin I argues against the possibility that the cortexillin imposes a distinct bending stiffness to the region bent around the negatively curved particle surface.

As a marker for curvature sensing in cortexillin I/II double-null cells, we monitored the accumulation of actin around the neck of budded yeast. In the cortexillin double-null cells, actin strongly accumulated at sites of negative curvature (see Additional file 6). We conclude that even under conditions of low bending stiffness of the cell cortex, curvature is recognized in phagocytic cups.

**Figure 8:**
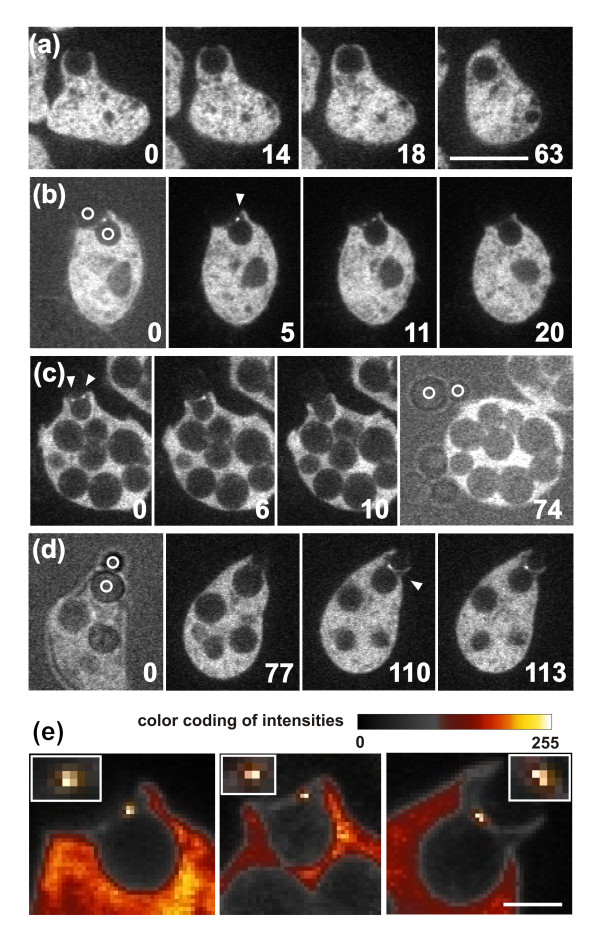
**Localization of I-BARa-GFP during yeast uptake**. **(a) **Uptake of an unbudded yeast. No accumulation of fluorescent IBARa at the cup that was formed around the spherical particle was detectable against the small, highly mobile clusters in the cytoplasm. **(b-d) **Partial uptake of budded yeast. Clusters of IBARa-GFP are indicated by arrowheads, positions of yeast mother cell and bud by circles. The IBARa protein disappeared from the neck region of the particle when uptake turned into release: 20 seconds in (b), and 10 seconds in (c). See Additional file 7 for the sequence shown in (d). In the 0 second images of (b, d) and the 74 seconds image of (c), the brightfield illumination is superimposed on the fluorescence image to show the budded yeast. **(e) **Quantification of fluorescence intensities of IBARa-GFP at the neck region of particles using a look-up table (color bar). Left panel, the cup in (b); middle panel, the cup in (c); right panel, the cup in (d). These images represent average projections over 10 frames (left), six frames (middle) or 14 frames (right) recorded at intervals of 0.5 seconds. For averaging, periods were selected in which the cup did not significantly move. Magnified regions of interest are shown in the inserts, demonstrating that the high fluorescence intensities are concentrated on about 2 pixels^2^. The pixel size is 168 × 168 nm. Time is indicated in seconds. Bar for (a-d) = 10 μm; Bar in (e) = 2 μm.

The second possibility we considered was that the cell might employ a geometric sensing mechanism, such that the negatively curved particle surface at the bud neck induces the binding of a protein containing an I-BAR domain. To probe for the capacity of IBARa (a *Dictyostelium *protein that contains both an I-BAR and an SH3 domain) to bind at the bud neck, we used an IBARa-GFP fusion. This protein was not detectably enriched during uptake of an unbudded yeast (Figure 8a), but became concentrated at the site of greatest negative curvature of a budded particle (Figures 8b to d and Additional file 7). Quantitative image analysis revealed that IBARa is localized to patches at the neck region of the particle which, regardless of their small size, are the brightest structures in confocal sections through the phagocytic cups (Figure 8e). Furthermore, the average projections shown demonstrate that the IBARa patches are bound at the neck region, persisting in the three examples for 4.5, 2.5 or 6.5 seconds (Figure 8e). Their endurance distinguishes these patches from the highly mobile IBARa aggregates that are responsible for the fluorescent background in the cytoplasm. The binding of IBARa proved to be connected to the uptake of a particle, as the release of a budded yeast was preceded by the disappearance of IBARa-GFP. Similarly, disassembly of actin filaments preceded the release of a particle (Figure 1; see Additional file 1). These data indicate that IBARa recognizes the outward bend of the phagosome membrane during the uptake of a particle, consistent with a role for this protein in coordinating the assembly of actin around a particle of complex shape.

### Localization of myosins during particle uptake and release

**Figure 9:**
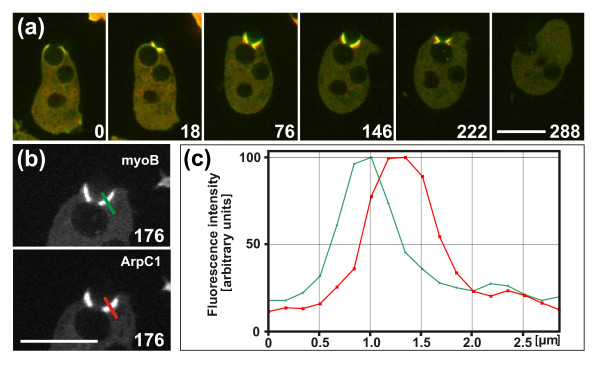
**Patterns of MyoB and of the Arp2/3 complex around the neck of a budded yeast**. **(a) **GFP-MyoB (green) and mRFP-ArpC1 (red) during partial uptake and release of the particle. The labels remained enriched for 3 minutes during arrest of the phagocytic cup at the neck of the particle and disappeared before the particle was released. For the complete series see Additional file 8. **(b) **One frame from the same time series as in (a), showing the fluorescences of GFP-MyoB (top) and mRFP-ArpC1 (bottom) in separate channels. The colored line indicates the scan position in (c). **(c) **Quantification of fluorescence distributions of MyoB (green) and ArpC1 (red) along the line scan denoted in (b). The highest values of fluorescence intensity are set to 100. Time is indicated in seconds after the first frame in (a). Bars = 10 μm.

The ability of a cell to apply force to the neck of a bi-lobed particle, together with the assembly of actin filaments at that site, suggests that actin-based motor proteins are involved in the response of the phagocyte to particles of complex shape. MyoB, known to be involved in phagocytosis [43], is concentrated in a layer close to the phagosome membrane and strongly accumulates around the neck region of the particle (Figure 9a; see Additional file 8). The localization of MyoB close to the membrane is evident when it is directly compared with that of the Arp2/3 complex at positions near to the neck of the particle (Figure 9b, c). The intense enrichment of MyoB at the neck during a severing attempt suggests that MyoB might play a role in force generation. This experiment also illustrates that after an unsuccessful severing attempt, both markers disappeared from the neck region and the yeast was released without any detectable involvement of MyoB or the Arp2/3 complex. This behavior contrasts with the exocytosis at the end of the phagocytic pathway, visualized using the same two markers (see Additional file 9).

**Figure 10:**
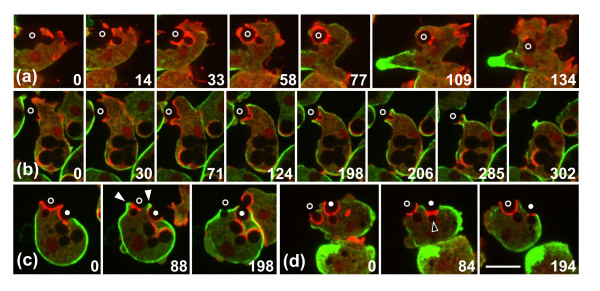
**Myosin-II engagement in particle release**. Cells interacting with budded yeast express GFP -myosin-II heavy chains (green) and the mRFP -LimEΔ label for filamentous actin (red). Positions of the yeast mother cells are indicated by open or closed circles. Time is indicated in seconds. **(a) **Complete uptake of a particle, showing no myosin-II associated with the phagocytic cup, except a slight enrichment at the plasma membrane when the phagosome is closing (109 seconds). The cell has a long tail rich in myosin-II, which is shown in full length during its retraction at 109 and 134 seconds. **(b) **Attempted uptake and release of a particle carrying a small bud pointing toward the cell. Myosin-II does not associate with the cup as long as it grows (30 and 71 seconds), but strongly accumulates during retraction of the cup and release of the particle (124 to 285 seconds). **(c) **Partial uptake of a yeast particle (open circles) showing no accumulation of myosin-II during extension of the cup (0 frame). Subsequently myosin-II is recruited to the plasma membrane surrounding the retracting cup (88 seconds, closed arrowheads), but even then not to the actin-rich neck region. Myosin-II accumulation is again observed during retraction of a second cup (closed circles). Finally, the second particle is engulfed by another cell, where myosin-II is again missing during cup extension (198 seconds). **(d) **Simultaneous release of one particle (closed circles) and partial uptake of another (open circles). Actin, which strongly accumulates at the neck of the first particle, is not accompanied by myosin-II (84 seconds, open arrowhead). Eventually, the second particle is also released. The enrichment of myosin-II in the retracting cups occurs asynchronously for the two particles. Bar = 10 μm for all images.

The question of whether the conventional, filament-forming myosin-II is engaged in the uptake of a particle was addressed using GFP-tagged myosin-II heavy chains [44]. Myosin-II proved not to be enriched at the phagocytic cup as long as the cup was extending, not even around the neck region of budded yeast (Figure 10a). Only at the end of uptake was myosin-II observed between the phagosome and the plasma membrane, consistent with a role in facilitating the closure of a cup or in driving the phagosome into the interior of the cell. However, when uptake failed and a particle was released, myosin-II was regularly recruited to the border of the retracting cups, consistent with its common function in cell contraction (Figure 10b to 10d; see Additional file 10 for another example).

**Figure 11:**
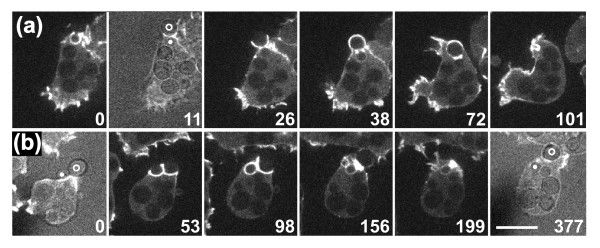
**Actin accumulation and severing of budded yeast in myosin-II-null cells**. In mutant cells deficient in myosin-II heavy chains, filamentous actin is labeled with LimEΔ- GFP. Time series showing **(a) **complete uptake with actin accumulation at the neck of the particle; **(b) **a case of severing. Brightfield images showing particle shape are superimposed on the fluorescence images at 11 seconds in (a) and at 0 and 377 seconds in (b). In these frames, the mother cell and bud of the yeast are denoted by circles and dots, respectively. Time is indicated in seconds. The complete series is shown in Additional file 11. Bar = 10 μm for all images.

Consistent with the absence of myosin-II from an extending phagocytic cup, cells lacking myosin-II heavy chains were able to take up yeast and did accumulate actin around the neck of a bi-lobed particle (Figure 11a). Moreover, we observed a myosin-II-null cell severing a budded yeast (Figure 11b; see Additional file 11), making it unlikely that myosin-II plays a role in generating the force required for severing.

## Discussion

### Decision making in particle uptake

To address the question of how a phagocyte recognizes a particle that can be engulfed by sealing the phagocytic cup around its end, we have exposed *Dictyostelium *cells to budded yeast particles, consisting essentially of two spheres connected by a concave neck. These living yeast correspond to particles that *Dictyostelium *cells encounter in their natural habitat. Our data show that the phagocyte has three choices when confronting a particle of complex shape: 1) cup extension can be stopped at a constriction of the particle to try to cut the particle there, allowing one portion to be engulfed; 2) cup extension may continue past the constriction until the entire particle is engulfed; 3) if severing is unsuccessful and the particle turns out to be too large for the cup to fully enclose it, the particle is released after a trial period.

These results extend the concept of a zipper mechanism governing phagocytosis. The zipper mechanism implies that a phagocytic cup progresses only as long as the actin polymerization machinery receives signals transmitted locally through the particle-attached membrane [45,46]. Studies in macrophages have linked phagocytic cup progression to the activities of Rho family GTPases [47]. Our results show that a cup not only stops growing if signaling ceases, but that different signal inputs are integrated, allowing the cup to switch between multiple modes of interaction with a particle surface. This flexibility is crucial for the cell response to a particle of complex shape. The switch from one behavior to the other can be fast, as seen in illustrations (Figure 2b; see Additional file 3) where actin stays for 4 minutes as a ring around the neck and thereafter redistributes within a 24-second period along the cup, which simultaneously resumes extension. The period required for actin disassembly coincides with the 25-second period calculated from FRAP for the turnover of filamentous actin at the neck region (Figure 4b).

The switch from uptake to release of a particle is essential in a natural environment to prevent the phagocyte from being incapacitated by particles that cannot be ingested because of their size or tight attachment to a surface. Potential mechanisms in the decision for release include the measurement of time elapsed during an unsuccessful phagocytosis attempt or measurement of the tension generated as a cell attempts to pull in an oversized particle [48].

Release of a particle before closure of the cup is preceded by the disassembly of actin filaments (Figure 1), suggesting an actin-based clamping mechanism that holds the particle within the cup. Adhesion of the particle to the phagocyte surface, although crucial for the initiation of a phagocytic cup, is apparently not sufficient to hold the particle within an open cup against the internal pressure of the cell. The disassembly of actin-based structures is also evident using MyoB and ArpC1 as markers (Figure 9; see Additional file 8). This disassembly distinguishes the mechanism of particle release after unsuccessful phagocytosis from that of exocytosis after phagosomal processing, which involves actin and associated proteins [49,50] (see Additional file 9).

### Ras activation and the turnover of actin structures at phagocytic cups

During the entire process of phagocytosis, the uptake system is in an unstable state that allows it either to focus on a concave neck or to progress further along the convex surface of a particle. The switching between different modes of action is made possible by the delicate balance of actin polymerization and depolymerization in a phagocytic cup. There is no question that actin is polymerizing at the edge of a growing phagocytic cup [51]. However, FRAP revealed that in phagocytosing *Dictyostelium *cells, the *de novo *polymerization of actin is not restricted to the cup's rim (Figure 4). Consistent with these data, Ras proved to be activated along the membrane of the entire bowl of the phagocytic cup (Figure 5). The local balance of actin polymerization and depolymerization can be visualized by the reversible recruitment of coronin (Figure 3), one of the regulatory proteins engaged in turning off actin polymerization [22]. Remote from the membrane, the actin layer was decorated with coronin (except at the growing edge of the cup), indicating that actin is being depolymerized at the interface between the actin layer and the cytoplasmic space. The conclusion is that along the entire surface of a phagocytic cup, the actin network is subject to continuous turnover during all stages of cup progression. As a result, phagocytosis is not a one-way event that either proceeds or stops, but a process in which positive and negative controls generate dynamic spatiotemporal patterns of activity that depend on the shape of the particle being engulfed.

### Patterns of actin assembly in phagocytosis

Analyzing the uptake of budded yeast enabled us to dissect signal transduction in phagocytosis into two pathways that guide the interaction of a cell with a particle of complex shape. In chemotaxis and spontaneous motility of *Dictyostelium *cells, actin polymerization is linked to the presence of PIP3 in the membrane [52,34]. Similarly, phagocytic cup formation depends on the local synthesis of PIP3 in response to the attachment of a particle [30,31]. In phagocytic cups containing budded yeast, no augmented accumulation of PIP3 in the neck region was detected (Figure 6a, b) nor were PI(4,5)P2 or PI(3)P elevated there (Figure 6c, d), indicating that the strong accumulation of actin at the neck is not primed by an underlying increase in any of these phosphoinositides in the membrane of the phagosome. Similarly, there was no evidence for stronger activation of Ras stimulating actin polymerization at the sites of negative particle curvature (Figure 5).

These results might be explained in the following ways. One possibility is that two signal systems compete with each other in the control of actin assembly during phagocytosis: one dependent on curvature and the other not. The curvature-independent system, involving PIP3 in cooperation with Ras, would stimulate expansion of the cup. By contrast, the curvature-dependent system would induce the strong accumulation of actin around the concave region of the particle's surface. Another possibility is that there is only a single system that regulates actin polymerization in response to particle shape, one that involves PIP3 and Ras. Previous data indicated that, even on a planar membrane, actin polymerization is enhanced at the sharp boundary of a PIP3-rich area [48], an effect that resembles the formation of a trigger wave in a bistable system [53]. Inactivation of Ras and depletion of PIP3 at the strongly curved membrane surrounding the neck of a particle are consistent with such a boundary effect (Figures 5 and 6). Any of these regulatory mechanisms of actin polymerization will require a sensing mechanism that distinguishes the negative curvature at the neck from the convex portion of a particle surface.

### Curvature recognition

We have considered two possibilities for curvature sensing that might be responsible for the strong accumulation of actin around the neck of a particle: 1) measurement of cortical tension at the bent membrane of a phagocytic cup and 2) recognition of membrane curvature by a protein containing an I-BAR domain [54-57]. To explore the first possibility, we used cortexillin I/II double-null mutants in which coupling of the cortical actin network to the plasma membrane is impaired, resulting in a dramatically reduced bending stiffness of the cell cortex [41]. In spite of this deficiency, the membrane of the mutant cells conformed closely to the concave neck of budded yeast, and actin filaments strongly accumulated there (see Additional file 6). Thus, stiffness of the cell cortex is unlikely to be a factor in shape recognition. In wild-type cells, cortexillin is uniformly depleted from phagocytic cups (Figure 7). The low bending stiffness of the cell cortex caused by this depletion may be relevant for the close apposition of the phagosome membrane to curved particle surfaces.

The enrichment of IBARa at the neck region of a particle indicates that localization of this I-BAR-containing protein is linked to the strongest negative curvature of the membrane, and suggests that it is involved in sensing the curvature. Through its SH3 domain, IBARa may act as an adaptor to recruit regulators of actin polymerization including the Arp2/3 complex, which strongly accumulates at the neck region of a particle. The SCAR/WAVE complex is unlikely to be a mediator of the strong polymerization of actin at the neck of a particle, because in SCAR-null mutants, actin still accumulates there (data not shown). A reasonable possibility would be RacC-mediated activation of WASP [58].

### Local versus global force generation

Previous work has implied that forces acting in different directions contribute to the uptake of a particle: a force that flattens the phagocytic cup and thus narrows the space between phagosome and plasma membrane, a force that causes contraction of the cup at its rim, and a force that pulls the particle into the cell [59,2]. The most prominent force generated during the uptake of budded yeast is directed against the neck of the particle. The flattening force does not appear to work in this context, as only the phagosome membrane and not the plasma membrane follows the negative curvature of the particle (Figure 2a, b). When a large particle is pulled into a cell, the cell cortex is elastically deformed and finally tension is built up [1]. The tension relaxes under two conditions: when the particle is completely engulfed [60], or when the actin layer of the phagocytic cup disassembles before the cup is closed and the particle is expelled [48].

The interaction of a *Dictyostelium *cell with a budded yeast is characterized by phases of expansion of the phagocytic cup and retraction to the concave neck of the particle (Figure 2). This alternation of distinct phases of activity is regulated at the level of the individual phagosome; it does not reflect phases of contraction and relaxation of the entire cell. This is evident when a phagocyte interacts simultaneously with two particles. In Figure 2b, the 79 to 449 second frames comprise an interval of 6 minutes in which the cup formed at one particle retracts, whereas the cup engulfing a second particle expands until this particle is completely enclosed. This local regulation of phagosome activities indicates that forces are generated independently at each phagosome in a spatial pattern dictated by the shape of the particle.

### Myosins in phagocytosis

Three classes of myosin have been implicated in phagocytosis by *Dictyostelium *cells: myosin VII, the conventional myosin-II capable of forming bipolar filaments, and single-headed type I myosins. Myosin VII-null mutants have deficiencies in adhesion to a particle [15]. The role of class II myosins appears to vary between different phagocytes and even between different types of uptake in the same phagocytes. In macrophages, inhibitor studies have implicated myosin-IIA in actin recruitment to phagocytic cups induced by the activation of complement receptor 3. However, in Fcγ-receptor mediated phagocytosis, myosin-IIA is involved only in a late step, possibly during closure of the cup [61]. In *Dictyostelium*, myosin-II is not required for the phagocytosis of bacteria [62] or for uptake of the larger yeast particles (Figure 11a). Furthermore, *Dictyostelium *mutants lacking the myosin-II heavy chain can accumulate actin at the neck of a budded yeast and sever the particle there (Figure 11b).

When considering the localization of myosin-II to phagocytic cups, it is crucial to distinguish the stage of the cup; that is, whether it is extending in an attempt to engulf a particle or regressing after a failed attempt. In phagocytosing *Dictyostelium *cells, myosin-II is not enriched at a growing cup, either around the concave neck or the convex portions of the particle. However, myosin-II is recruited to the border of a retracting cup, a behavior reminiscent of its association with the contracting tail of a migrating cell (Figure 10; see Additional file 10). Recruitment to retracting cups may account for the report of myosin-II at phagocytic cups and surrounding the neck of budded yeast particles [14], as that study examined fixed and immunolabeled *Dictyostelium *cells, in which the status of the cups could not be determined.

*Dictyostelium *cells express12 unconventional myosins. Seven isoforms of myosins-I have partially overlapping functions [63-65]. Of these myosins, MyoK, MyoC and MyoB have been localized to the constriction of a phagocytic cup [14] (Figure 9). There they may act in concert to generate force. MyoB was the first myosin shown to be involved in phagocytosis [43]. This 'long-tailed' myosin-I possesses a tail homology TH2 domain, which binds to actin, and an SH3 domain, which binds to the Arp2/3 complex through the linker protein CARMIL (capping, Arp2/3, myosin I linker protein homolog) [66]. MyoK has been proposed in a recent report to form a circuit with Abp1-PakB that regulates the uptake efficiency of large particles [14]. These findings point to the importance of type 1 myosins in phagocytosis. We suggest below how these myosins might act to produce a constricting force.

### Distinct mechanisms for contraction of a cleavage furrow and constriction of a phagocytic cup

The constriction of a phagocytic cup at its rim and around the neck region of a particle is distinguished from contraction of the cleavage furrow in mitotic cells by the proteins involved. This is particularly clear in *Dictyostelium*. Whereas myosin-II is recruited to the cleavage furrow, it is absent from constrictions of the phagosome as long as particle uptake proceeds (Figure 10). Similarly, cortexillin is accumulated in the cleavage furrow and is important for cytokinesis in *Dictyostelium *[40,67], but is depleted in phagocytic cups (Figure 7).

**Figure 12:**
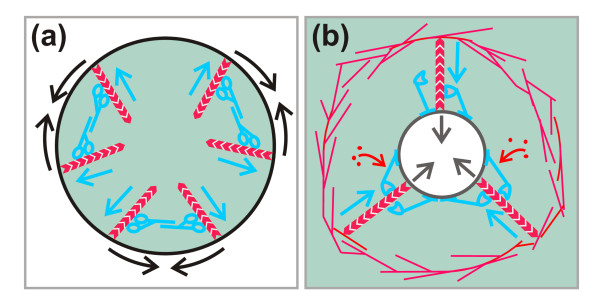
**Diagram of proposed myosin-I action in phagocytic cup constriction compared with myosin-II function in the contraction of a cleavage furrow**. **(a) **Schematic cross-section through a cleavage furrow. Contraction of the cleavage furrow in mitotic cells is reinforced by the conventional myosin-II that forms bipolar filaments with the motor domains pointing into opposite directions (blue). In this simplified scheme, actin filaments (red chevron polymers) attached with their (+) ends to the plasma membrane are assumed to point into the cytoplasmic space of the cleavage furrow, as suggested previously [77]. By moving in the (+) end direction (blue arrows), the myosin-II filaments apply force on the perimeter of the cleavage furrow, thus causing it to contract. **(b) **Schematic view of the orifice at the rim of a phagocytic cup at the neck of a budded yeast particle being engulfed (black circle). Actin filaments are again assumed to point from the plasma membrane into the cytoplasmic space. They act against a barrier of branched and cross-linked actin filaments in the cell cortex. Clusters of membrane-bound myosin-I molecules (blue) move by their motor domains in the (+) end direction (blue arrows) and are proposed to allow actin subunits to enter. The force applied against the barrier is postulated to cause the orifice of the bud to constrict or the neck of a particle to be severed (black arrows).

The diagrams in Figure 12 illustrate in a simplified manner the principal difference between the two contraction mechanisms, that at a cleavage furrow and that at a phagocytic cup. At a cup, myosins-I such as MyoB and MyoK are capable of linking, through their lipid-binding or farnesylation sites, the actin network to the phagosome membrane. With their motor activity, these myosins may push growing actin filaments away from the membrane against the dense network of Arp2/3-associated filaments. Force generation by growing actin filaments is consistent with the high polymerization rate of actin at the phagosome membrane surrounding the neck region of a particle (Figure 4). MyoB has been demonstrated to act as a force sensor [68], and thus would be capable of holding actin filaments in place in a force-dependent manner. A likely role for MyoB in phagocytic force generation is further emphasized by its importance in *Entamoeba histolytica*; in this pathogenic phagocyte, MyoB is the only unconventional myosin present [28].

## Materials and methods

Transformants of *D. discoideum *strain AX2-214 were cultivated in petri dishes at subconfluent densities at 23 ± 2°C in nutrient medium containing selective agents (G418 and blasticidin) for maintenance of the plasmids. For phagocytosis experiments using living budded yeast, cells of *Saccharomyces cerevisiae *strain TH2-1B were cultivated overnight at 30°C as described previously [23]. This yeast strain with the genotype *MATa mnn1 mnn2 *is a mutant of X2180, altered in the structure of cell wall mannoproteins. The cell shown in Figure 1 happened to lack vacuolin B, which is of no relevance here.

### Vector construction and cell transformation

#### PH-PLCδ1-GFP

The plasmid PLC-δ1 pEGFPN1 [69] was used as template for PCR to generate *Asp*718 sites at the N- and C-terminal ends of the coding sequence of the pleckstrin homology (PH) domain of human phospholipase Cδ1. The resulting PCR product was cloned into pGemT easy (Promega Corp., Madison, WI, USA). The PH-PLCδ1 fragment was excised with *Asp*718 and cloned into the *Asp*718 site of the *Dictyostelium *expression vector pTXGFP [70]. The correct orientation was determined by sequencing. Plasmid PH-PLCδ1-GFP was introduced by electroporation into *D. discoideum *cells and transformants were selected with G418 (10 μg/ml Geneticin; Gibco BRL Life Technologies Inc., Grand Island, NY, USA).

#### IBARa-GFP

The full-length open reading frame of the *ibrA *gene(DictyBase ID: DDB_G0274805) was amplified from cDNA using primers with *att*B GateWay recombination sites http://www.invitrogen.com and the PCR product cloned into the GateWay entry vector pDONR221. The PCR product was confirmed by sequencing. For GFP fusion, the *ibrA *gene was cloned into the GateWay destination vector pDM450 [71].

#### GFP-Raf1-RBD and GFP-NdrC-RBD

A minimal RBD comprising amino acid residues 55 to 131 of human Raf protooncogene serine/threonine protein kinase (Raf-1) served as an activation-specific probe for Ras [72,73]. The nucleotide sequence of a gene fragment encoding the human Raf-1 RBD adapted to the *D. discoideum *codon usage was synthesized (Eurofins MWG Operon, Huntsville, MA, USA) and cloned via *Bam*HI and *Eco*RI downstream of GFP into a pDEX-based expression vector [74]. Transformants of the AX2-214 strain of *D. discoideum *were selected using 10 μg/ml blasticidin.

Alternatively, the Ras-binding domain of the AGC-kinase NdrC from *D. discoideum *(gene number DDBG0284839) (Weeks and Müller-Taubenberger, unpublished data) was used to probe for activated Ras. A cDNA encoding amino acid residues 2 to 300 was cloned via *Bam*HI into the GFP expression vector. To relate actin accumulation to Ras activation, GFP-Raf1-RBD or GFP-NdrC-RBD was combined with mRFP-LimEΔ, which was expressed under selection of G418 (10 μg/ml).

### Confocal fluorescence microscopy

For microscopic observation, *D. discoideum *cells in the exponential phase of growth were transferred to a chamber consisting of a plastic ring (19 mm inner diameter, 4 mm height) that had been attached to a cover glass with paraffin wax. Once the cells had settled, the nutrient medium was replaced with phosphate buffer (17 mM KH_2_PO_4_/Na_2_HPO_4 _buffer, pH 6.0). After about 30 minutes, yeast were added. Within one hour, excess yeast were removed, and the cells were overlaid with a thin layer of agarose [75]. The chamber was covered with a second cover glass held in place with silicone grease.

Most confocal time-lapse sequences were captured by an Ultra View ERS system (Perkin-Elmer LAS Inc., Norton, OH, USA) linked to a TE2000 microscope (Nikon Instruments Inc., Tokyo, Japan) equipped with a 100× objective (Plan-Apochromat VC; numerical aperture (NA)1.4; Nikon Instruments Inc.). Images were acquired at 0.5, 1 or 2 second intervals; and GFP and mRFP were excited sequentially with the 488 and 568 nm laser lines, respectively. Emission was detected through a triple dichroic and a double band-pass emission filter on an electron multiplying charge coupled device (EMCCD) camera. Figure 5 was recorded on a spinning disc microscope (Olympus/Andor, Avon, MA, USA) with a 60× oil objective (PlanApoN/NA 1.42) as described previously [76]. For the presentation of fluorescence intensities in Figure 8e, the software ImageJ 'smart' lookup table (LUT) http://rsb.info.nih.gov/ij/ was used.

For the experiments shown in Figures 1, 2, 3(a) and 6(a, b), images were collected using a confocal microscope (LSM510; Zeiss, GmBH Jena, Germany) equipped with a 63× differential interference contrast objective (Plan-Apochromat oil, 1.4 NA; Nikon Instruments Inc.). Images were acquired at 3.94 second intervals. S65T-GFP was excited with the 488 nm line of an argon laser with a 505 to 530 nm filter for emission, and mRFP was excited with the 543 nm line of a HeNe laser, with a 560 or 585 nm long-pass filter for emission. A UV/488/543/633 main beam splitter was used.

### FRAP experiments

Fluorescence recovery of GFP-actin and mRFP-LimEΔ was determined (UltraView ERS 6 system; Perkin Elmer) with a photokinesis unit for photobleaching. For bleaching and image acquisition, a 60×/NA 1.4 oil objective (Nikon Instruments Inc.) was used with a pinhole size of Airy 2 to integrate a large depth of focus. The phagosomes were kept in focus by gently overlaying the cells with an agarose sheet.

For bleaching, a circular region of interest of 6 μm in diameter was selected. The first post-bleach images were taken <500 ms after the bleaching pulse. The 488 nm laser used bleached GFP and to a lesser extent mRFP. GFP and mRFP images were acquired sequentially at a rate of 1 frame/second. The channel settings for GFP and mRFP were, respectively, 488 and 561 nm for excitation, 527/70 and 615/70 nm for emission. An EM CCD camera (C9100-50; Hamamatsu Photonics, Hamamatsu, Japan) minimized bleaching during image acquisition. In Figure 4(b, c) the low background of fluorescence measured within the phagocytic cup has been subtracted. The bleaching of GFP-actin during the period of imaging proved to be negligible; the mRFP-LimEΔ curves were corrected for a bleaching rate of 0.008/second, as measured in the cytoplasmic area of a control cell. For Figure (4d to 4f) the Image J 'fire' LUT http://rsb.info.nih.gov/ij/ was used.

## Competing interests

The authors declare that they have no competing interests.

## Authors' contributions

MC contributed transformants and performed most of the experiments. UE performed the FRAP experiments. JG first recorded the severing of budded yeast. AM-T contributed fluorescent RBD constructs for the localization of activated Ras. JP performed phagocytosis experiments and analyzed data. DV generated the IBARa-GFP construct used for localization of the I-BAR protein. GG participated in experiments and coordinated the work.

## Supplementary Material

Additional file 1 **Movie 1 - Attack of two budded yeast by a cell expressing LimEΔ-GFP as a label for filamentous actin**. The fluorescence is superimposed in green on greyscale brightfield images. Protrusions surrounding the particle are fluctuating between forth and back movement. This movie shows the same events as Figure 1. Frame to frame interval is 4 seconds. All Additional files are movies showing phagocytes of *Dictyostelium discoideum *that interact with living yeast particles. Bars in the movies indicate 10 μm. Click here for file

Additional file 2 **Movie 2 - Different modes of interaction with budded yeast**. The cell expresses PHcrac-GFP (green), a label for phosphoinositides PI(3,4,5)P3 and PI(3,4)P2 and mRFP-LimEΔ (red) for actin filaments. The fluorescence images are superimposed on greyscale brightfield images. This sequence, corresponding to Figure 2a shows cup extension, retraction to the neck, and severing of the particle. Frame to frame interval is 4 seconds. All Additional files are movies showing phagocytes of *Dictyostelium discoideum *that interact with living yeast particles. Bars in the movies indicate 10 μm. Click here for file

Additional file 3 **Movie 3 - Reversible switching between cup extension and retraction**. The labels are the same as in Movie 2. This sequence corresponds to Figure 2b. Frame to frame interval is 4 seconds. All Additional files are movies showing phagocytes of *Dictyostelium discoideum *that interact with living yeast particles. Bars in the movies indicate 10 μm. Click here for file

Additional file 4 **Movie 4 - Coronin dynamics relative to actin polymerization and depolymerization**. The cell taking up a budded yeast particle expresses GFP-coronin (green) and mRFP-LimEΔ (red). The movie is shown twice: first with the fluorescent labels superimposed on the greyscale brightfield images, subsequently without the brightfield images. During cup extension, coronin localizes to the boundary of the actin layer bordering the cytoplasm. After the completion of uptake, coronin persists during the period of actin disassembly. Images from this sequence are presented in Figure 3a. Frame to frame interval is 2 seconds. All Additional files are movies showing phagocytes of *Dictyostelium discoideum *that interact with living yeast particles. Bars in the movies indicate 10 μm. Click here for file

Additional file 5 **Movie 5 - FRAP of GFP-actin at the neck region of budded yeast and during the extension of a phagocytic cup**. The sequence corresponds to Figure 4(d, e). Brightfield illumination is interspersed between the fluorescence images to visualize the position of the particle. Frame to frame interval is 1 second. All Additional files are movies showing phagocytes of *Dictyostelium discoideum *that interact with living yeast particles. Bars in the movies indicate 10 μm. Click here for file

Additional file 6 **Movie 6 - Actin accumulation at the neck region of budded yeast taken up by mutant cells that are deficient in both cortexillin I and II**. Complete uptake is shown in the left cell and attempted uptake in the right cell. Frame to frame interval is 1 second. All Additional files are movies showing phagocytes of *Dictyostelium discoideum *that interact with living yeast particles. Bars in the movies indicate 10 μm. Click here for file

Additional file 7 **Movie 7 - Accumulation of IBARa-GFP at the neck region of a budded particle**. This sequence corresponds to Figure 8d. It shows progression of a phagocytic cup over the neck region and final release of the particle. I-BARa is concentrated for a period of 42 second at the neck, before it disappears there prior to release of the particle. Frame to frame interval is 0.5 seconds. All Additional files are movies showing phagocytes of *Dictyostelium discoideum *that interact with living yeast particles. Bars in the movies indicate 10 μm. Click here for file

Additional file 8 **Movie 8 - Localization of GFP-MyoB (green) and mRFP-ArpC1 (red) to the phagosome membrane surrounding the neck of a particle**. Intense accumulation of these markers persists for 3 minutes, and release of the particle is preceded by their disappearance. Frame to frame interval is 1 second. All Additional files are movies showing phagocytes of *Dictyostelium discoideum *that interact with living yeast particles. Bars in the movies indicate 10 μm. Click here for file

Additional file 9 **Movie 9 - Release of a yeast carcass at the end of the phagocytic pathway**. This sequence shows the involvement of GFP-MyoB (green) and mRFP-ArpC1 (red) during exocytosis. Frame to frame interval is 1 second. All Additional files are movies showing phagocytes of *Dictyostelium discoideum *that interact with living yeast particles. Bars in the movies indicate 10 μm. Click here for file

Additional file 10 **Movie 10 - Recruitment of GFP-myosin-II to a retracting cup**. The cell expresses GFP-myosin-II heavy chain (green) and mRFP-LimEΔ (red). Myosin-II does not colocalize with actin at the neck of the particle, but localizes to the lateral border of the cup during its retraction, and to other retracting regions of the cell body. Frame to frame interval is 1 second. All Additional files are movies showing phagocytes of *Dictyostelium discoideum *that interact with living yeast particles. Bars in the movies indicate 10 μm. Click here for file

Additional file 11 **Movie 11 - A myosin-II-null cell that severs a budded yeast particle**. The cell expressing GFP-LimEΔ is also shown in Figure 11b. Brightfield images are interspersed between the fluorescence recordings to show the particle before and after severing. Frame to frame interval is 1 second. All Additional files are movies showing phagocytes of *Dictyostelium discoideum *that interact with living yeast particles. Bars in the movies indicate 10 μm. Click here for file
